# Optimizing Ergonomic Practices in Radiology: A Closed-Loop Audit of Workstation Standards and Staff Wellbeing

**DOI:** 10.7759/cureus.99149

**Published:** 2025-12-13

**Authors:** Shivangi Yadav, Chiranji Lal Goel

**Affiliations:** 1 Internal Medicine, York and Scarborough Teaching Hospital NHS Foundation Trust, Scarborough, GBR; 2 Radiology, Jindal Institute of Medical Sciences, Hisar, IND

**Keywords:** digital eye strain, musculoskeletal pain, occupational health service, radiologist well-being, workstation ergonomics

## Abstract

Introduction: Radiology professionals are increasingly exposed to prolonged computer use, placing them at risk for musculoskeletal, ocular, and postural health issues. Studies report that up to 88.9% of radiologists experience work-related musculoskeletal disorders, while 36-50% report ocular strain or visual fatigue. Optimising workstation ergonomics is therefore critical for maintaining both staff wellbeing and diagnostic accuracy.

Aim: To assess compliance with the Royal College of Radiologists (RCR) ergonomic workstation standards among radiology staff and evaluate improvements following targeted interventions between audit cycles.

Methods: A two-cycle electronic questionnaire audit was carried out, with 46 responses in total - 25 in 2023 and 21 in 2024 - from consultant radiologists and reporting radiographers across two hospital sites. Audit standards were based on the RCR ergonomic guidance - “Correct Use of Reporting Workstation.” The questionnaire was developed from this publicly available RCR standard and adapted for local use. Data were analysed using descriptive statistical methods to summarise and compare compliance between the two audit cycles.

Results: Overall, 100% (25/25 in 2023 and 21/21 in 2024) had access to voice recognition in both cycles, and there was an increase from 9/25 (36%) to 14/21 (67%) in terms of variable lighting at workstations. Eye test compliance declined in the second cycle of the audit, and only 10/21 (48%) reported taking regular breaks, which improved from 9/25 (36%) since the first cycle. Moreover, awareness of ergonomic guidelines improved, but conformance remained low.

Conclusion: While improvements were noted in some domains, gaps remain in key areas such as break-taking and regular eye tests. Sustained efforts are required to support ergonomic practice adoption and reduce staff health risks.

## Introduction

Ergonomics in the radiology workspace encompasses workstation design and work patterns that account for the health, comfort, and efficiency of clinicians. While technical optimisation of radiology software systems, such as picture archiving and communication systems (PACS), has advanced considerably, ergonomic considerations have often lagged behind. The increasing reliance on digital reporting and the extended screen time required for image interpretation have heightened awareness of work-related health risks, particularly musculoskeletal disorders (MSDs), repetitive strain injuries, and visual fatigue among radiologists and reporting radiographers [1-3].

The Royal College of Radiologists (RCR) emphasises that optimal workstation setup, adequate lighting, and regular breaks are critical in maintaining performance and minimising fatigue [4]. Poor ergonomic compliance can lead not only to discomfort and reduced job satisfaction but also to diagnostic inaccuracies, as prolonged static posture and visual strain are known to impair attention and perception [3,5-8]. Previous studies have reported that radiologists frequently experience neck, back, and shoulder pain related to workstation setup and prolonged sedentary work, underscoring the need for evidence-based ergonomic interventions in radiology departments [3,6,7].

Recognizing these risks, the RCR issued standards to guide workstation design, promote awareness of safe working practices, and encourage regular review of individual and environmental factors influencing reporting quality. However, despite these recommendations, compliance remains variable across institutions, often influenced by workload, awareness, and resource availability.

Aim: This audit aimed to assess compliance with the RCR ergonomic workstation standards within radiology reporting rooms across two hospital sites in a UK NHS Trust and to evaluate changes following targeted interventions over a one-year period.

Audit standard: All staff undertaking radiology reporting should comply with the RCR ergonomic recommendations, which include having regular eye tests, taking scheduled breaks, using voice-recognition dictation, and having access to adjustable seating and variable ambient lighting at workstations.

Criteria for re-audit success: Demonstration of improved compliance rates of 100% for eye tests, regular breaks, voice-recognition dictation, adjustable seating, and variable ambient lighting, and 85% compliance for postural assessment.

## Materials and methods

This audit was conducted within two radiology departments across two different sites of a single NHS Trust in the UK. The project was registered with the Trust’s Research and Development Department (CSCS/RAD/022) and conducted in two cycles over a period of a year, from 31/10/2023 to 8/11/2024. The project was done to evaluate adherence of the RCR standards of "Correct Use of Reporting Workstation" [4].

Inclusion criteria are all consultant radiologists and reporting radiographers actively engaged in diagnostic image reporting. Eligible participants were required to use a PACS workstation as part of their routine reporting activity to ensure adequate exposure to ergonomic factors. Only fully completed survey responses were analysed.

Exclusion criteria included administrative staff and other personnel not performing independent image reporting, as well as staff on extended leave (e.g., maternity, sickness, or career break) during the audit cycle. Incomplete or partially completed survey responses were also excluded.

A cross-sectional electronic questionnaire survey titled “Ergonomics in Radiology” was distributed to all eligible consultant radiologists and reporting radiographers across both sites via Microsoft Forms. A total of 46 completed responses were received - 25 in 2023 and 21 in 2024. The questionnaire was adapted from the RCR standards of “Correct Use of Reporting Workstation” and included nine questions addressing key ergonomic domains, including compliance with eye testing, use of voice recognition software, weekly reporting hours, break-taking habits, awareness of health and safety executive (HSE) posture guidelines, posture-related health consultations, and workstation environment factors such as lighting (Table 1).

**Table 1:** Electronic questionnaire.

No.	Question	Response
1	What is your job role?	Consultant Radiologist/Reporting Radiographer
2	Have you had an eye test within the last two years?	Yes/No
3	Are you able to utilise voice recognition dictation?	Yes/No
4	How much time do you spend at your workstation per week?	<5 hrs/5–10 hrs/10–20 hrs/20–30 hrs/>30 hrs
5	Do you take regular breaks away from your workstation (e.g., 5 minutes every hour)?	Yes/No
6	Are you aware of the HSE Good Posture Guidelines?	Yes/No/Unaware of guidelines
7	If you are aware of the Good Posture Guidelines, does your workspace conform to them?	Yes/No
8	Have you seen your GP/physiotherapist for an issue relating to your posture at your workstation?	Yes/No
9	Do you have variable lighting at your workstation?	Yes/No

Responses were collected anonymously during two audit cycles (2023 and 2024) and analysed in Microsoft Excel (Microsoft® Corp., Redmond, WA). Data were summarised as absolute numbers and percentages (N, %). Compliance levels were compared between the two cycles to assess progress and identify persistent gaps. Descriptive analysis was considered appropriate as this was a service evaluation aimed at measuring adherence to standards rather than testing a research hypothesis.

The target compliance levels were defined according to the “Correct Use of Reporting Workstation,” which recommends 100% compliance for eye tests, regular breaks, voice-recognition dictation, adjustable seating, and variable ambient lighting, and 85% compliance for postural assessment. This threshold was used to evaluate whether departmental performance met expected standards of ergonomic compliance.

Between the first and second audit cycles, several targeted interventions were implemented to address identified gaps. These included ergonomic teaching sessions for radiology staff, display of HSE posture posters and RCR ergonomic guidelines in reporting areas, departmental upgrades to improve variable ambient lighting, and reinforcement of regular breaks and micro-breaks during departmental meetings. Ergonomics was also incorporated into routine teaching, with plans for re-audit to assess the sustainability of improvements.

Participation was voluntary, and not all invitees responded, which accounted for the modest sample size. This approach ensured voluntary engagement and ethical data collection. As the data were self-reported, a minor risk of recall or response bias was recognised.

## Results

**Figure 1:**
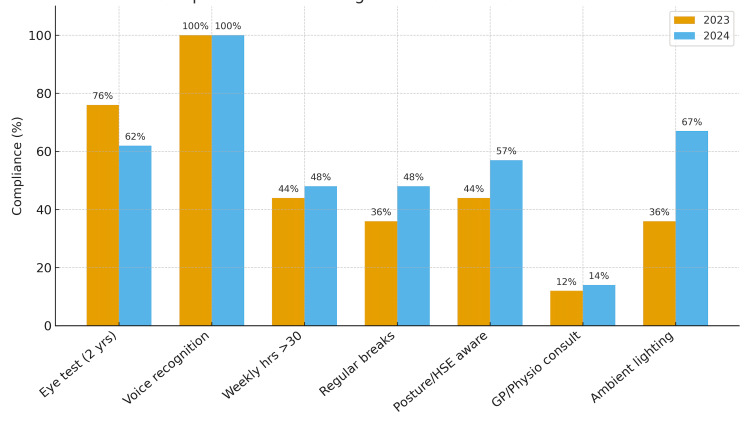
Comparison of ergonomic compliance between 2023 and 2024. The clustered bar chart illustrates compliance rates across key ergonomic domains. The X-axis represents ergonomic standards, and the Y-axis indicates percentage compliance (0–100%).

The 2024 re-audit demonstrated mixed progress when compared with 2023 (Figure 1). The most notable improvement was access to variable ambient lighting, which increased from 9/25 (36%) in 2023 to 14/21 (67%) in 2024 - a 31% increase, reflecting a significant enhancement in the ergonomic work environment. Uptake of regular reporting breaks improved from 9/25 (36%) to 10/21 (48%), a 12% increase, and awareness of posture and HSE guidelines rose from 11/25 (44%) to 12/21 (57%), a 13% improvement. Staff reporting over 30 weekly workstation hours rose modestly from 11 of 25 respondents (44%) in 2023 to 10 of 21 (48%) in 2024, a 4% relative increase largely attributed to the smaller denominator in 2024, despite a reduction in absolute numbers. Importantly, full compliance with voice recognition software access was maintained across both audit cycles (25/25 (100%) vs. 21/21 (100%) (Table 2).

**Table 2:** Data analysis.

Standards	2023	2024	Change	Comments
Eye test within last 2 years	19/25 (76%)	13/21 (62%)	↓ 14%	Compliance dropped below target
Voice recognition software access	25/25 (100%)	21/21 (100%)	-	Maintained full compliance
Weekly reporting hours (>30 hours/week)	11/25 (44%)	10/21 (48%)	↑4% *	Percentage appears higher due to smaller denominator, absolute number slightly decreased
Takes regular reporting breaks	9/25 (36%)	10/21 (48%)	↑ 12%	Growing awareness
Aware of posture/HSE guidelines	11/25 (44%)	12/21 (57%)	↑ 13%	Knowledge not translating into behaviour
Consulted GP or physio for posture-related issue	3/25 (12%)	3/21 (14%)	↑ 2%	Slight increase, possible ongoing ergonomic strain
Access to variable ambient lighting	9/25 (36%)	14/21 (67%)	↑ 31%	Significant improvement in environmental setup

Conversely, compliance with recommended biennial eye testing fell from 19/25 (76%) to 13/21 (62%), representing a 14% decline and dropping below the audit target. Furthermore, despite improved awareness of ergonomic guidance, behavioural translation appeared limited, as evidenced by a slight rise in consultations with general practitioners or physiotherapists for posture-related issues (3/25 (12%) vs. 3/21 (14%)). Percentage interpretation should be viewed cautiously, given the smaller 2024 sample size (n = 21 vs. 25 in 2023), which may exaggerate proportional differences despite similar absolute counts.

## Discussion

This audit highlights the continuing challenge of embedding ergonomic good practice in radiology, where awareness, behaviour, and departmental priorities are often misaligned. The observed improvements in lighting and voice-recognition systems likely reflect departmental equipment upgrades rather than individual behavioural change. In contrast, areas requiring personal initiative - such as taking regular breaks or scheduling eye tests - showed lower compliance, suggesting that heavy workload, time pressure, and entrenched reporting culture remain key barriers. At our Trust, lighting upgrades coincided with reading room refurbishment, whereas the decline in eye test compliance may reflect limited protected time for non-clinical appointments.

Previous research supports these findings, with several studies demonstrating that poor ergonomic compliance and extended reporting time are associated with increased musculoskeletal and visual strain among radiologists [2,3,6]. The modern digital radiology environment - with its constant image flow, remote reporting, and performance targets - promotes long, uninterrupted sessions at workstations. Such patterns reinforce sedentary postures and visual fatigue, both linked to musculoskeletal discomfort and reduced diagnostic accuracy [7-9]. Krupinski et al. demonstrated that extended reporting hours impair lesion detection and visual accommodation, supporting the value of regular “micro-breaks" [8]. However, the culture of productivity over rest persists, making consistent ergonomic behaviour difficult even among aware staff.

Similar findings have been reported elsewhere. Rodrigues et al. found that, although most radiologists understood ergonomic principles, few adjusted their workstations or sought occupational advice because of time pressure and lack of institutional facilitation [6]. Alelyani et al. likewise identified prolonged PACS use and high workload as key predictors of musculoskeletal symptoms [3]. These studies, together with our findings, confirm that education alone is insufficient - sustained compliance depends on leadership engagement, workflow planning, and departmental support.

Embedding ergonomics within routine departmental processes could help shift responsibility from individuals to systems. Initiatives such as scheduled micro-break reminders, regular workstation checks, ergonomic induction sessions, and annual eye care prompts could normalise safe practices. Following the dissemination of this audit, ergonomic awareness was included in departmental teaching, and a re-audit is planned to assess progress. As Soussahn et al. highlighted, ergonomics should be recognised as a core component of diagnostic quality assurance, essential for both accuracy and staff wellbeing [2].

Limitations

This audit has several limitations. The findings are based on self-reported data, which may not accurately reflect actual ergonomic behaviours. The sample size was modest and limited to a single NHS Trust, reducing the generalisability of the results. Data collection was descriptive rather than inferential, so changes between cycles cannot be interpreted as statistically significant. Some questions required subjective self-assessment, such as awareness of posture guidelines, which may vary among respondents. The audit also focused primarily on compliance with ergonomic recommendations rather than objective outcomes such as diagnostic accuracy or sickness absence. Finally, factors such as workload, individual workstation variability, and previous ergonomic training were not assessed, and these may have influenced responses.

Recommendations

To address the remaining gaps, the department plans to continue ergonomic teaching, reinforce break-taking reminders, integrate regular eye-check prompts, and maintain re-audit cycles to ensure sustained improvement.

## Conclusions

This two-cycle audit demonstrated measurable but incomplete improvement in ergonomic compliance among radiology staff. Environmental factors, including improved lighting and sustained voice recognition access, achieved high compliance, reflecting effective departmental changes. However, behavioural domains - such as regular breaks and routine eye testing - showed limited progress, indicating that awareness did not consistently translate into practice. These results highlight persistent barriers related to workload, time pressure, and departmental culture. The audit therefore met its primary aim of identifying progress and ongoing gaps against the RCR ergonomic standards, providing a baseline for further improvement. Continued monitoring through re-audits will help assess the impact of implemented measures and ensure sustained staff wellbeing and diagnostic accuracy.
